# Supplementary material for: Development and Validation of a Rapid Lateral Flow E1/E2-Antigen Test and ELISA in Patients Infected with Emerging Asian Strain of Chikungunya Virus in the Americas
Source: Viruses. 2020 Sep 1;12(9):971. doi: 10.3390/v12090971 (PMC7552019; doi:10.3390/v12090971)
Supplement: Supplementary file 1 [file viruses-12-00971-s001.zip › Supp Fig Legends.docx]

**Table S1: Limits of Detection of Antibody Pairs to detect CHIKV.** The limits of detection (LoD) and dissociation constant (Kd) were calculated for antibody Combination A (48 and 155) for the dipstick, lateral flow, and ELISA format using decreasing concentrations of E1 and E2. LoD and Kd was calculated for antibody Combination B (4 and 340) for the lateral flow format using decreasing concentrations of CHIKV virus-like particles (VLP).

**Figure S1:** **Binding of CHIKV antibodies.** Monoclonal antibody clones were harvested from the hybridomas of CHIKV-immunized mice. The antibodies were then screened through ELISA to analyze the binding with Chikungunya virus-like particles (VLP) and the genetically-related Mayaro VLP. The OD_450_ signal is represented as fold above background.

**Figure S2:** **Binding of CHIKV FACS Positive Clones.** Following screening of antibodies through ELISA, 16 out of the 41 ELISA positive clones were positive using FACS analysis with CHIKV vero infected cells. The OD_450_ signal is represented as fold above background for the FACS positive clones.

**Figure S3: Combinatorial Dipstick Analysis of CHIKV Clones.** Functional antibody pairs were defined through combinatorial dipstick trials. One antibody was conjugated to gold nanoparticles and one antibody was adsorbed to nitrocellulose membrane. The resulting nanoparticle conjugates-membrane pairs were tested using the CHIKV virus-like particles (VLP) and MAYV VLP as a counter-screen. These proteins were present at a concentration of 150 ng/ml in the testing. The signal on the dipstick is measured on a scale from 0 (no visible signal) to 1 (strongest signal) to measure antibody sandwich binding to VLP.

**Figure S4: Limit of Detection of Antibody Pairs to detect CHIKV.** Limits of Detection (LoD) were

calculated using either Combination A (48 and 155) or Combination B (4 and 340) antibodies. The LoDs  for Combination A were measured in dipstick format (A-B), lateral flow format (C-D), and ELISA (E-F), all by measuring the normalized signal intensity with decreasing concentrations of CHIKV E1 and E2 proteins. The LoD was measured for Combination B in a lateral flow format using decreasing concentrations of CHIKV virus-like particles (VLP) (G).
