# Supplementary material for: Development and Validation of a Rapid Lateral Flow E1/E2-Antigen Test and ELISA in Patients Infected with Emerging Asian Strain of Chikungunya Virus in the Americas
Source: Viruses. 2020 Sep 1;12(9):971. doi: 10.3390/v12090971 (PMC7552019; doi:10.3390/v12090971)
Supplement: Supplementary file 1 [file viruses-12-00971-s001.zip › TableS1andLegend.docx]

**Table S1: Limits of Detection of Antibody Pairs to detect CHIKV.** The limits of detection (LoD) and dissociation constant (K_d_) were calculated for antibody Combination A (48 and 155) for the dipstick, lateral flow, and ELISA format using decreasing concentrations of E1 and E2. LoD and K_d_ was calculated for antibody Combination B (4 and 340) for the lateral flow format using decreasing concentrations of CHIKV virus-like particles (VLP).

|  | **Dipstick** | **Lateral Flow** | **Lateral Flow** | **ELISA** |
| --- | --- | --- | --- | --- |
| **Antibody Pair** | 48; 155 | 48; 155 | B.1; B.2 | 48; 155 |
| **E1 LoD (ng/mL)** | 15.55 | 844.16 |  | 133.48 |
| **E1 Kd** | 125.81 | 2402.61 |  | 400.43 |
| **E2 LoD (ng/mL)** | 37.08 | 875.86 |  | 363.98 |
| **E2 Kd** | 202.15 | 299.79 |  | 1091.93 |
| **VLP LoD (ng/mL)** |  |  | 76.90 |  |
| **VLP Kd** |  |  | 78.46 |  |
