# Supplementary figures and images for: Development and Validation of a Rapid Lateral Flow E1/E2-Antigen Test and ELISA in Patients Infected with Emerging Asian Strain of Chikungunya Virus in the Americas
Source: Viruses. 2020 Sep 1;12(9):971. doi: 10.3390/v12090971 (PMC7552019; doi:10.3390/v12090971)

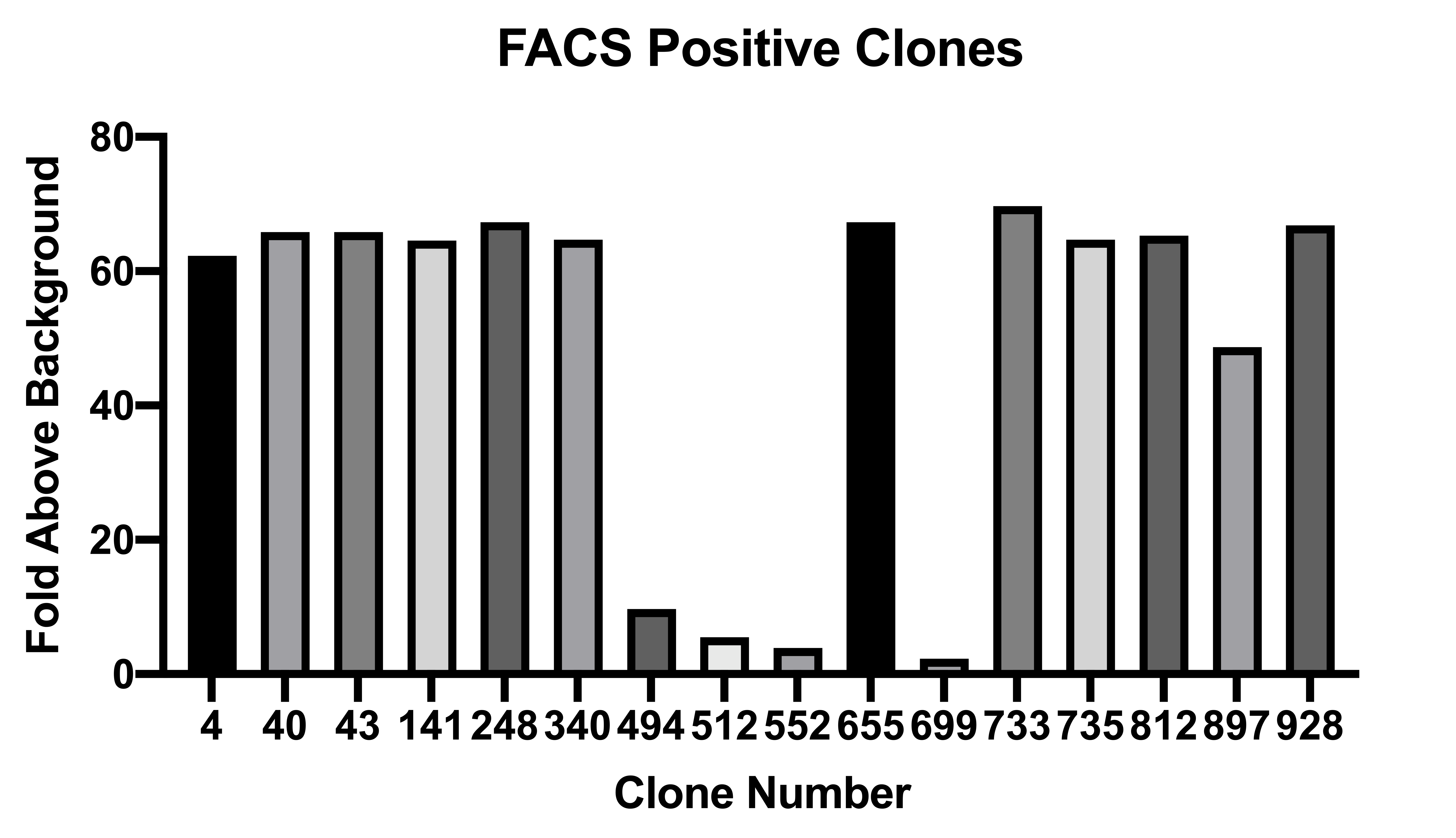

Supplement: Supplementary file 1 [file viruses-12-00971-s001.zip › SuppFig2 (1).tiff]

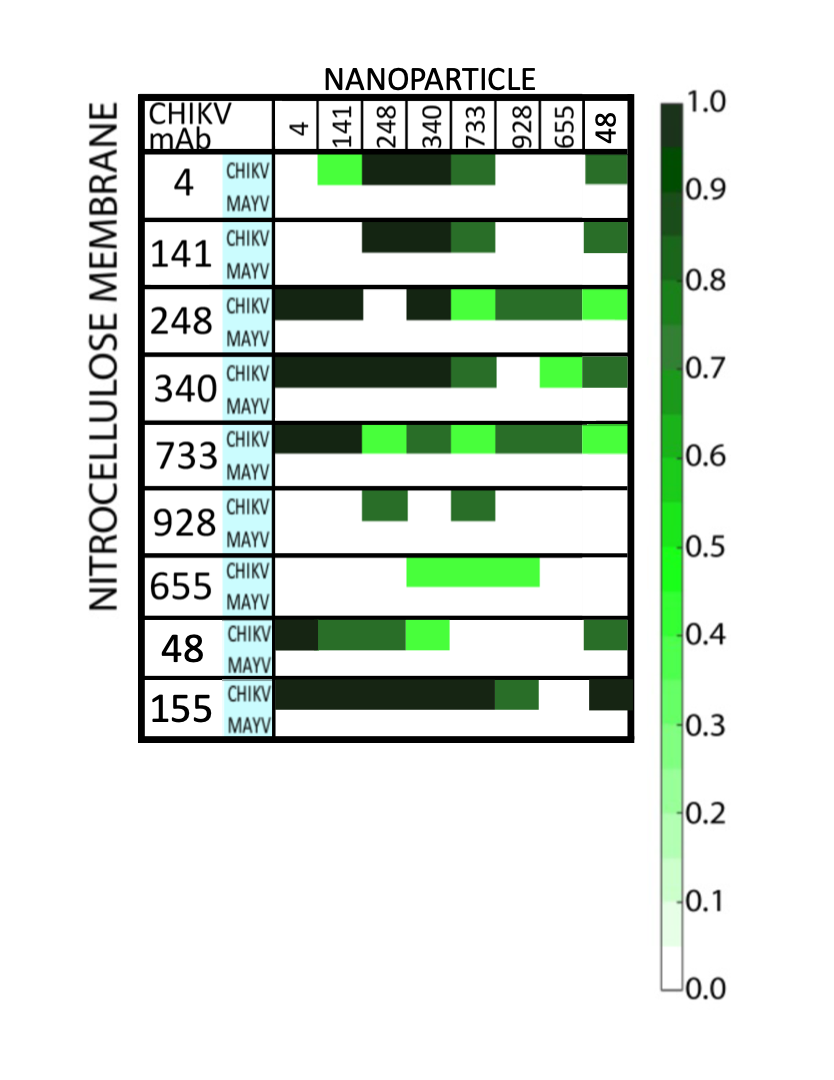

Supplement: Supplementary file 1 [file viruses-12-00971-s001.zip › SuppFig3.png]

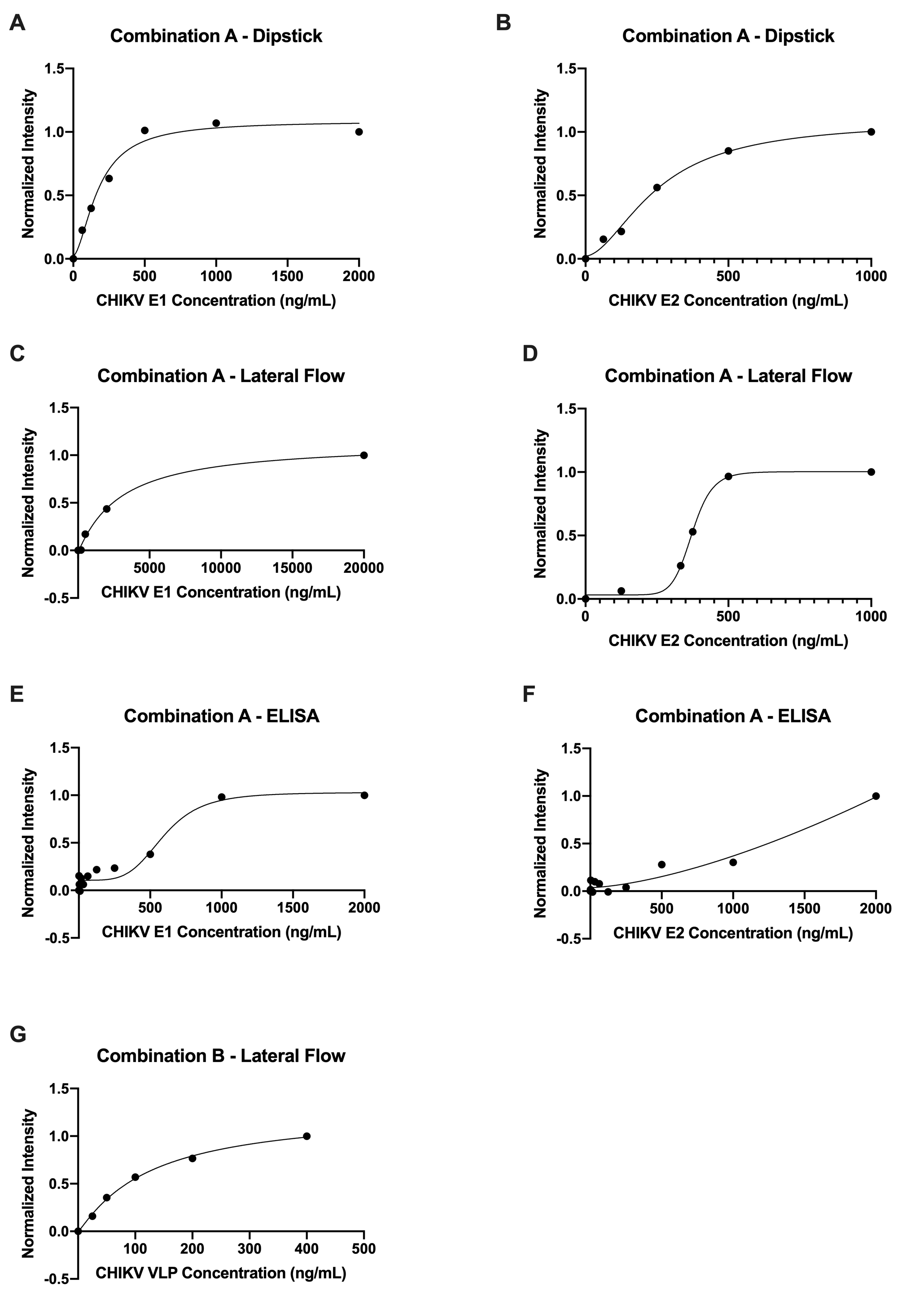

Supplement: Supplementary file 1 [file viruses-12-00971-s001.zip › SuppFig4 (1).tiff]
